# Newly Qualified Professional Nurses' Readiness for the Independent Practice Role

**DOI:** 10.1155/nrp/6876561

**Published:** 2025-03-07

**Authors:** Warriodene Hansen

**Affiliations:** Department of Health Studies, University of South Africa, Pretoria, South Africa

**Keywords:** guidance, newly qualified professional nurse, preceptorship, readiness, support, transition

## Abstract

**Background:** Newly qualified professional nurses find the transition to their new role difficult and need support to connect theory to practice. The readiness to their new role is identified with stress, fear, lack of confidence, anxiety and lack in clinical ability. It was clear that newly qualified professional nurses need further academic and clinical support from the clinical institutions they are employed at. The challenges of their new role transition are assigned to various factors, amongst others, needing more preparation, finding it difficult to bridge the theory–practice gap and having no guidance or support.

**Aim:** The aim of this study was to explore the professional transition experiences of newly qualified professional nurses to their new role.

**Setting:** The research setting in this study was two hospitals in the Cape Winelands region of the Western Cape, South Africa.

**Methods:** This study employed a descriptive phenomenological research design. The researcher collected data through interviews. The data were analysed by employing a thematic analysis method.

**Results:** Three themes were discovered from the findings of this study, with subthemes supporting them. Inadequate support was found to be a cause for not being prepared. Consequently, newly qualified professional nurses blame peers for lack of support.

**Conclusion**The participants of this study all described that newly qualified professional nurses' transition to nursing was challenging. The participants agreed that the transition to the professional role is a concern that needs to be addressed. This study found a need for professional support and guidance of newly qualified professional nurses within the clinical environment.

## 1. Introduction

Newly qualified professional nurses find the transition to their new role difficult. This descriptive phenomenological study explored the professional transition experiences of newly qualified professional nurses to the independent practice role. Being in at the deep and following the increased responsibilities of their new role and changing identities, from student to newly qualified professional nurse, are off the factors found which are challenging during the transition period [1] as well as, stress, theory to practice confusion and an increased risk for errors, making it difficult for newly qualified professional nurses to adapt to their role [2]. These experiences are often due to the lack of overseeing and help, as can be presented by a preceptor [3].

In the Republic of South Africa, newly qualified professional nurses face many challenges while being new to the practice role [4]. Therefore, a structured programme should be established to help ease the transitioning of these newly qualified nurses and implemented once the newly qualified nurses start to practice refining their clinical competence [5]. In 2011, the South African Department of Health recommended the establishment of a clinical teaching unit in which nursing education institutions would employ preceptors to accompany nursing students during their clinical placements [6].

## 2. Problem Statement

Various authors confirm that newly qualified professional nurses find the transition to their new role difficult and need support to connect theory to practice [7]. In South Africa, newly qualified professional nurses perform compulsory community service, and during this period, they require support, guidance and supervision of experienced professional nurses to ensure a seamless transition into the workplace [8]. Clinical support and guidance in a nurses' education plays a vital role in preparing them for their role as competent, able and caring nurses [9]. This prompted the researcher to explore the transition experience of newly qualified professional nurses towards their independent practice role to utilise the data to develop a preceptorship model for newly qualified professional nurses.

## 3. Research Objectives

The following research objectives were formulated:

• To explore how newly qualified professional nurses experienced the transition towards independent practice role.
• To explore what constitutes the development of a preceptorship model for newly qualified professional nurses.

## 4. Research Design and Methods

### 4.1. Research Design

This study utilised a descriptive phenomenological design. This design was beneficial in exploring and describing participants' experiences. Also, it allowed for the collection of information about factors that could assist with developing a suitable preceptorship model for newly qualified professional nurses.

### 4.2. Description of the Characteristics of Descriptive Phenomenology

The researcher utilised the key characteristics of Husserl's phenomenology, description, reduction, essence and intentionality as follows.

#### 4.2.1. Description

This is the peak of descriptive phenomenological research where a description or model presents the phenomenon under study to a degree that those who experienced the phenomenon can see their own experience [10]. The researcher achieved this through writing a thesis to share the research results and participants' experiences and synthesise the research study.

#### 4.2.2. Reduction

Once the results were analysed, then only the description of the results was performed, aided with literature control.

#### 4.2.3. Essence

Essence is capturing experience right from the beginning, without interpretation and explanation [11]. In this study, an interview guide was utilised. The meaning of the data was given by generating essential themes.

#### 4.2.4. Intentionality

In this view, the researcher took a subjective stance to seek the real experiences of participants, hence employing semistructured interviews.

### 4.3. Steps of Descriptive Phenomenological Research

Descriptive phenomenological research includes bracketing, intuiting, analysing and describing [12]. These steps were applied as follows.

#### 4.3.1. Bracketing

Data were collected based on the research objectives and questions. During data collection and analysis, the researcher kept observational notes as a reflective journal to write down reactions and experiences and did not share personal experiences to prevent biases.

#### 4.3.2. Intuiting

This was achieved by the researcher reflecting on the participants' experiences and remaining open to understanding the preceptorship as shared by participants.

#### 4.3.3. Analysing

Analysis was conducted to discover themes, character and categories to quote noteworthy accounts, and through this, the researcher made sense of the essential meaning of the phenomenon of this study. This process was backed by illustrating the findings within the original data rather than the researcher's understanding. An experienced phenomenological researcher familiar with the phenomenon under study was appointed as a cocoder.

#### 4.3.4. Describing

Data were presented based on rich narrative descriptions of transition experiences from the newly qualified professional nurses. At this stage, the researcher described and defined the findings once it was understood and looked for any commonalities.

### 4.4. Study Population

The population was all the nurses employed on a full-time basis at the two public hospitals. The first was a public regional hospital, who receives referrals from the primary healthcare facilities, district hospitals within the region and the local community. The other research setting, a public tuberculosis hospital, also provides a service to the district within the region it is located.

### 4.5. Sampling

Purposive sampling was applied to achieve the research objectives. Purposive sampling is a sampling method in which the researcher purposefully selects participants or includes selection elements [13]. The participants were purposefully selected to get rich information about the professional transition of newly qualified professional nurses. The participants were selected based on a set of inclusion and exclusion criteria.

### 4.6. Data Collection

Data were collected by means of semistructured interviews utilising an interview guide. The key informers in this study were newly qualified professional nurses. Participants were given the freedom to respond to the research questions. Field notes were utilised for reflection of the interview process.

### 4.7. Data Analysis

A thematic analysis method, utilising Braun and Clarke's [14] six-step thematic analysis method, was applied for data analysis. The following data are organised and prepared for analysis:

• After the recorded interviews were transcribed, it was read by the reseacher to become familiar with the information and data.
• Next, the researcher commenced with coding of the data.
• Then, themes were generated to represent the data.
• Themes and subthemes were checked by reviewing the codes and ensuring that the combinations of the codes form a coherent pattern.
• The initial thematic maps were adjusted where needed. Finally, the researcher ascertained that the themes reflected the data.
• The themes were analysed to ensure they represent the underlying data and the participants' experiences.
• A robust descriptive research report of the findings was compiled.

## 5. Ethical Considerations

Ethics is determining right and wrong and exploring what to do when confronted with a situation where values, rights, personal beliefs or societal norms may be in conflict [15]. The participants' rights were upheld as follows.

### 5.1. Respect for Persons

This study received ethical approval from the University of South Africa's Institutional Review Board, College of Human Sciences Research Ethics Review Committee, reference number: Rec-2408 16-052 on 23 March 2021. Gateway permission was obtained from the Western Cape Department of Health and the nursing managers of the respective hospitals. All participants signed informed consent and participated voluntarily. The researcher ensured privacy and confidentiality by assigning participant numbers to participants during transcribing and data analysis. All methods were carried out in accordance with relevant guidelines and regulations as set within the Declaration of Helsinki.

### 5.2. Beneficence

The major benefit of the research was shared with the participants. No interviews were stopped or rescheduled because of participants experiencing discomfort or emotional distress.

### 5.3. Justice

The researcher provided sufficient information to the participants and reminded them that they can withdraw from this research without any explanation. The newly qualified professional nurses were vulnerable individuals, and the researcher ensured that they were not targeted for the researcher's convenience to participate but to contribute to the development of the preceptorship model.

## 6. Measures to Ensure Trustworthiness

The trustworthiness of the data obtained was measured against the four constructs, namely, credibility, transferability, dependability and confirmability [16].

### 6.1. Credibility

Credibility was enhanced by describing and discussing the findings and applying current literature into the arguments. Furthermore, the researcher had consensus meetings with an intercoder, who holds a PhD in nursing, to correlate and discuss between the codes and findings discovered, followed by a consensus meeting.

### 6.2. Transferability

Transferability involves the quality of a report that its findings can be applied to other contexts and settings. This was ensured through thick description of the two settings as well as the profile of the participants and the methods followed in conducting the study, to enhance the chances of the study being repeated by other researchers. The researcher wrote a report describing the results in depth using verbatim quotations from the interviews, with the findings supported by literature control, after analysis.

### 6.3. Dependability

The individual interviews were audio-recorded to document the findings and back up the data and will be kept for 5 years as an audit trail.

### 6.4. Confirmability

The participants' perspectives were shared in the findings with direct quotations from the transcribed interviews. Peer debriefing ensured confirmability.

## 7. Results

Newly qualified professional nurses described their transition to the professional role as not being prepared well enough to perform independently. In addition, Benner's Novice to Expert theory's main premise is that newly qualified professional nurses need support and guidance to be safe and competent nurses ([17], p. 1). Together with readiness for the role, the newly qualified professional nurses lacked in clinical competency.

However, newly qualified professional nurses were perceived as being responsible and accountable for their actions, especially a shift leader now. They have a goal to improve ([18], p. 24). According to Hansen and Zuma [19], newly qualified professional nurses are perceived as to be pushed in the deep end and their task can be difficult at times. This is due to the inherent role of the professional nurse, such as being a mentor to students while not long ago they were students themselves. Adding to this is that they find their their clinical tasks difficult at times. They need guidance offered by a colleague with more experience ([20], p. 2931). Their undergraduate clinical learning exposure is insufficient, and this adds to the problem of clinical ability. Thus, a clinical learning programme would improve newly qualified professional nurses' clinical challenges. However, this requires ongoing career development, and to achieve this, the newly qualified professional nurse goes through various stages in the acquisition of clinical skills ([21], p. 402).

Important with new implementation within a department, the ongoing support of nurse management is vital because it relates to higher job satisfaction ([22], p. 374). According to Hansen and Zuma [23], the operational managers in this study perceived the newly qualified professional nurses' readiness for the professional role as a desire to be clinically independent and increasing their clinical ability. They described that there were factors which influenced the newly qualified professional nurses' readiness for the professional role, such as the new clinical environment and how they perceive their new role. Although being graduates, the operational managers described them as still needing support in the clinical environment [24]. Significantly, off Kolb's learning cycle of experiential learning's main premise is that knowledge is created through experience ([25], p. 41). However, the newly qualified professional nurses should open to learn and have a personal drive to succeed.

Three themes were discovered from the findings of this study, with subthemes supporting them. The results of this study are discussed per participant group. A total of 25 participants participated in this study.  Table 1 contains the demographic information of the participants.

The age mode of newly qualified professional nurses was under 30 years, and all newly qualified professional nurses were females (*n* = 11; 100%). Of the newly qualified professional nurses, 8 (73%) held a degree in nursing, while 3 (27%) held a diploma in nursing. The majority of newly qualified professional nurses were assigned in medical areas (*n* = 6; 55%) and (*n* = 2; 18%) in intensive care, (*n* = 1; 9%) orthopaedics, (*n* = 1; 9%) obstetrics and gynaecology and (*n* = 1; 9%) emergency unit.

### 7.1. Theme and Subthemes

The theme that emerged was preparedness for the role and had five subthemes. First, the study's major findings were discussed and interpreted based on literature control to put the findings in context, which entails the newly qualified professional nurses transition experience, together with the research objectives and research questions of this study. The data are presented in themes and subthemes. The themes that emerged from the thematic analysis are presented in Table 2.

#### 7.1.1. Theme 1: Preparedness for the Role

Prepared to perform the role of a newly qualified professional nurse was discovered as a theme in connection with the transition experiences of newly qualified professional nurses towards nursing. The participants shared not being prepared well enough to act as a newly qualified professional nurse. Together with preparedness, participants were not ready for the professional nurse's role. Contrary to the participants having negative experiences towards the transition to the independent practice role, some of the participants had positive experiences had positive experiences ranging from being a student to being a newly qualified professional nurse. The following subthemes provide a comprehensive view of the experiences of the newly qualified professional nurses.

##### 7.1.1.1. Subtheme: The Transition of the Newly Qualified Professional Nurse

This subtheme emerged based on the transition experiences encountered by the newly qualified professional nurses. These experiences include feeling unprepared for the independent practice role, unsure and being dependent. This can be emphasised as expressed by the participants: ‘My taught theory compared to what I see now is different, because I don't feel prepared for what I am experiencing in practice now' as stated by a female newly qualified professional nurse, 24 years old. Other participants shared the same experiences, as expressed: ‘When I started, I was not prepared yet' another female newly qualified professional nurse, 23 years old, stated.

Newly qualified professional nurses shared that being newly engaged they were scared and nervous. They found this period being stressful and difficult, while the pressure of being newly appointed newly qualified professional nurses also influenced their experiences. They seemed unsure and dependent, as expressed: ‘The first time when I walked in here, I was very scared because of what was expected from me and what I felt my knowledge was, I was scared' another female newly qualified professional nurse, 32 years old, stated. During their transition, they found it very difficult to adapt to their new role as well as grasping theory with practice.

##### 7.1.1.2. Subtheme: Working Environment

The working environment is where the transition was experienced. The newly qualified professional nurses would find it difficult to adapt to their assigned department or unit. They experienced a lack of support and guidance from the experienced professional nurses. As expressed, ‘Professional nurses that are working here are experienced, they are supposed to guide us, but they find it also very difficult to guide and support us and make them us feel we can ask questions' another female newly qualified professional nurse, 28 years old stated. Thus, most significant was their dependency on their colleagues, most of the time staff nurses.

##### 7.1.1.3. Subtheme: Performing the Role

Adapting to a new role was challenging to the newly qualified professional nurses. The scope of a professional nurse and its duties was perceived as being a leader and as a big responsibility, experienced as expressed

*‘I had a few shortcomings especially personally uhm with my confidence and dealing with people or the staff members or all the staff members'* another female newly qualified professional nurse, 28 years old stated.

Another said

*‘Uhm there is still those moments where you doubt yourself but then you realize you, you are in charge. Okay, you are in charge of a ward because everyone will come to you if something is wrong'* a female newly qualified professional nurse, 23 years old, stated.

##### 7.1.1.4. Subtheme: Professional Support and Guidance

All newly qualified professional nurses shared their experiences on how they managed their clinical ability in order to feel independent, gain confidence and competence. They solved their own problems, some had been helped from peers and some stated they were independent, knowledgeable and skillful, as expressed

*‘I could work on my own'* another female newly qualified professional nurse, 28 years old stated. The participant further said: ‘*I could work on my own, I had more experience even though it was my first year, I had more experience because uhm as a student, I usually found a Professional Nurse that was my mentor'.*

##### 7.1.1.5. Subtheme: Motivation to Perform Role

Wanting to succeed and perform well in their new role was very important to the participants. They never gave up or wanted to quit but were willing to learn, as expressed

*‘I won't say I was competent but most of the stuff I knew what to do; the more I was expose to a thing, the eager I got to learn more'* a female newly qualified professional nurse, 25 years old, stated.

Another said

*‘…personal experience, like a certain situation will happen in the ward and maybe goes wrong but then you know, okay tomorrow when this happens, I know exactly how to handle this situation uh, so you also learn through your mistakes as well, with experience. As you experience certain situations with things, you also learn with that'* a female newly qualified professional nurse, 22 years old, stated.

## 8. Discussion

Not receiving adequate support, for example from their peers, seemed to be a major reason for newly qualified professional nurses in this study not being prepared well enough. Additionally, not having a dedicated clinical teacher was a contributing factor to the newly qualified professional nurses' readiness. This study highlights the importance of ongoing guidance of newly qualified professional nurses by means of clinical teaching by experienced professional nurses.

To describe newly qualified professional nurses' experiences of developing competence, it was found that even if newly qualified professional nurses are willing to learn, they find it difficult to develop their professional competence [26]. Importantly, in this study, the newly qualified professional nurses found their transition challenging when applying theory to practice, but they were eager to become independent. As highlighted by Brady [27], they must accept their new assigned role and being held accountable must be an inherent characteristic. Furthermore, in this study, the newly qualified professional nurses described that they felt protected by their student status when it came to working by themselves.

The new clinical environment was overwhelming, but receiving help from colleagues eased the newly qualified professional nurses settling into their new workplace. Yet there were colleagues that were unwilling to help. This study found that newly qualified professional nurses had to get familiar with routines of their new clinical areas and what they learned as a student did not always correspond to the clinical environment. This was the case as well in a study where newly qualified professional nurses followed the routines of the respective work areas although it did not respond to the undergraduate education [26]. There was a lack of ability especially in performing their role that is inherent to a professional nurse, such as responsibility, delegation and leadership. This study found that motivation for newly qualified professional nurses was intrinsic. Therefore, it is important for clinical units to ensure ongoing clinical growth by creating an environment to improve clinical ability [28].

### 8.1. Implication for Nursing Practice

The findings contributed to the development of a model that has an outcome of readiness and preparedness by the newly qualified professional nurses. In addition, this study found the following practical phases to be exercised within the clinical area.

According to Hansen and Zuma [23], during the initial phase, newly qualified professional nurses are experiencing transition within the clinical environment. The transition to their new role has its challenges and paves the starting of the preceptorship when newly qualified professional nurses arrive as novices with limited experience. The preceptorship follows set objectives to reach its end goal. To reach the set objectives, a variety of clinical teaching and learning activities are planned, before determining readiness and preparedness to the professional role. Newly qualified professional nurses' competency is determined through assessment of the learning that would take place.

Overall, the results could have beneficial practical implications, as the preceptorship model created during the study can be used in clinical settings during newly qualified professional nurses' transition period and with any newly qualified person's learning activity. Also, this model may guide operational managers and preceptors in the effective preceptorship of newly qualified professional nurses and clarify their role within the preceptorship process. The findings may influence policymakers such as nursing governing bodies to incorporate the preceptorship model for newly qualified professional nurses as a prerequisite to qualifying as a professional.

## 9. Conclusion

This study highlights that while adapting to their new role, newly qualified professional nurses were unsure, not ready for the professional nurse's role and still dependent. It was agreed that the professional transition period of newly qualified professional nurses was a concern that needs to be addressed. This study found that a formal intervention is necessary to help newly qualified professional nurses during this period. The data collected in this study add to the development of a preceptorship model based on the experiences of the newly qualified professional nurses adding positive contributions to them becoming competent and independent, thus ensuring that they receive guidance and support from both preceptors and nursing managers who collaborate to achieve an effective preceptorship [23]. With a competent clinical teacher and the personal drive, the transition period can be a more pleasant experience, where both the clinical institutions and newly qualified professional nurses can have success. Importantly, a preceptorship model for newly qualified professional nurses might reduce the challenges and realities experienced.

## 10. Limitations

This study employed a qualitative approach by using a nonprobability sampling technique, limiting the generalising of the findings.

## 11. Recommendations

It is recommended to conduct a study to determine the effectiveness of support and guidance by means of a preceptorship for newly qualified professional nurses.

It is recommended that preceptorship is implemented and supported by nursing management.

## Figures and Tables

**Table 1:** Demographic information of participants.

Question	Category	*n*	%
What is your age?	Under 30	9	82
	31–40 years	2	18

What is your gender?	Male	0	0
	Female	11	100

Where is your current area of assignment?	Medical	6	55
	Obstetrics and gynaecology	1	9
	Orthopaedics	1	9
	Emergency unit	1	9
	Intensive care	2	18

What is your highest professional qualification?	Degree	8	73
	Diploma	3	27

**Table 2:** Themes produced.

Themes	Subthemes
1. Preparedness for the role	1.1. The transition of newly qualified professional nurses 1.2. Working environment 1.3. Performing the role 1.4. Professional support and guidance 1.5. Motivation to perform role

## Data Availability

The author confirms that the data supporting the findings of this study are available within the article.
